# Inhibition of NADPH Oxidase (NOX) 2 Mitigates Colitis in Mice with Impaired Macrophage AMPK Function

**DOI:** 10.3390/biomedicines11051443

**Published:** 2023-05-14

**Authors:** Suhrid Banskota, Huaqing Wang, Yun Han Kwon, Jaya Gautam, Sabah Haq, Jensine Grondin, Gregory R. Steinberg, Waliul I. Khan

**Affiliations:** 1 Farncombe Family Digestive Health Research Institute, McMaster University, Hamilton, ON L8S 4L8, Canada; banskots@mcmaster.ca (S.B.); wanghu@mcmaster.ca (H.W.); yyoon90@gmail.com (Y.H.K.); shaq@unmc.edu (S.H.); grondij@mcmaster.ca (J.G.); 2 Department of Pathology and Molecular Medicine, McMaster University, Hamilton, ON L8S 4L8, Canada; 3 Centre for Metabolism, Obesity and Diabetes Research, McMaster University, Hamilton, ON L8S 4L8, Canada; gautamj@mcmaster.ca (J.G.); gsteinberg@mcmaster.ca (G.R.S.); 4 Department of Medicine, McMaster University, Hamilton, ON L8S 4L8, Canada; 5 Department of Biochemistry and Biomedical Sciences, McMaster University, Hamilton, ON L8S 4L8, Canada

**Keywords:** macrophages, AMPK, NOX2, inflammation, autophagy, colitis

## Abstract

Macrophage adenosine monophosphate-activated protein kinase (AMPK) limits the development of experimental colitis. AMPK activation inhibits NADPH oxidase (NOX) 2 expression, reactive oxygen species (ROS) generation, and pro-inflammatory cytokine secretion in macrophages during inflammation, while increased NOX2 expression is reported in experimental models of colitis and inflammatory bowel disease (IBD) patients. Although there are reductions in AMPK activity in IBD, it remains unclear whether targeted inhibition of NOX2 in the presence of defective AMPK can reduce the severity of colitis. Here, we investigate whether the inhibition of NOX2 ameliorates colitis in mice independent of AMPK activation. Our study identified that VAS2870 (a pan-Nox inhibitor) alleviated dextran sodium sulfate (DSS)-induced colitis in macrophage-specific AMPKβ1-deficient (AMPKβ1^LysM^) mice. Additionally, VAS2870 blocked LPS-induced TLR-4 and NOX2 expression, ROS production, nuclear translocation of NF-κB, and pro-inflammatory cytokine secretion in bone marrow-derived macrophages (BMDMs) from AMPKβ1^LysM^ mice, whereas sodium salicylate (SS; AMPK β1 activator) did not. Both VAS2870 and SS inhibited LPS-induced NOX2 expression, ROS production, and pro-inflammatory cytokine secretions in bone marrow-derived macrophages (BMDMs) from wildtype (AMPKβ1^fl/fl^) mice but only VAS2870 inhibited these effects of LPSs in AMPKβ1^LysM^ BMDMs. Furthermore, in macrophage cells (RAW 264.7), both SS and VAS2870 inhibited ROS production and the secretion of pro-inflammatory cytokines and reversed the impaired autophagy induced by LPSs. These data suggest that inhibiting NOX2 can reduce inflammation independent of AMPK in colitis.

## 1. Introduction

Inflammatory bowel disease (IBD) is a chronic relapsing condition of the gastrointestinal tract that encompasses two conditions, namely Crohn’s disease (CD) and ulcerative colitis (UC). Although the exact etiology of IBD is unknown, it has been so far understood that multiple factors, such as immune dysregulation, dysbiosis, and environmental factors, contribute to the initiation and perpetuation of intestinal inflammation [1,2,3,4,5]. At present, various approaches, such as immunosuppressive chemotherapy, biologics, a combination of biologics and chemotherapy, and surgery, are utilized to manage IBD [6,7]. However, the adverse effects associated with the long-term use of biologics and chemotherapies urge the discovery of a novel target to achieve greater beneficial effects while mitigating adverse events in treating intestinal inflammation.

Macrophages play a key role in the generation of immune responses and inflammation in the gastrointestinal tract. Previous studies suggest that human intestinal macrophage subsets and mouse intestinal macrophages show similarities in homeostasis and inflammation [8]. Several investigations have outlined the possibility of generating drugs targeting macrophages, which, by exploiting their phagocytic ability, may enhance efficacy and reduce adverse effects in treating conditions such as IBD [9]. 

Adenosine monophosphate-activated protein kinase (AMPK) is an αβγ-heterotrimer that regulates cellular metabolism and requires the catalytic α-subunit and the regulatory β- and γ-subunits in order to carry out its physiological functions. Activating AMPK in macrophages using berberine, salicylates, or the AMPK β1-specific activator A769662 has been effective in inhibiting bacterial lipopolysaccharides (LPSs) or palmitate-induced inflammation [10,11]. Previously, we demonstrated that both 5-amino salicylic acid (Mesalazine) and sodium salicylate (SS) exert beneficial effects in dextran sodium sulfate (DSS)-induced intestinal inflammation through activation of macrophage-specific AMPK β1 complexes [4], emphasizing the important role of macrophage AMPK in the regulation of intestinal inflammation. 

Toll-like receptors (TLRs) are pattern recognition receptors with various homologous subtypes, including TLR4, which is a well-known endotoxin receptor for LPSs. The inhibition of TLR4-mediated NF-κB nuclear shuttling has been implicated in the downregulation of cyclooxygenase-2 (COX-2) and suppression of the production of inflammatory cytokines, such as tumor necrosis factor (TNF)-α, Interleukin (IL)-1β, and IL-6, in IBD patients [12]. Previous studies suggest the role of TLR4/NADPH oxidase (NOX) 2 in LPS-stimulated macrophages’ phagocytic and bactericidal functions [13,14]. NOX is a group of transmembrane enzymes comprising multiple membrane-bound and cytosolic subunits among which the prototype NOX2 is mainly expressed by immune cells, including macrophages [15]. NOX2 produces reactive oxygen species (ROS) after stimulation, which are a microbicide and an important signaling molecule in various cellular functions, such as cell growth, differentiation, and regulation of the immune response [15,16]. Studies have demonstrated that inhibition of NOX2 activity in mice by using Diphenyleneiodonium chloride (DPI) and VAS2870 (a pan-NOX inhibitor) reduced DSS-colitis in mice, and downregulated pro-inflammatory cytokine secretion in LPS-induced RAW264.7 cells [5,17]. Similarly, activation of AMPK has been reported to suppress NOX2 activity by inhibiting the nuclear translocation of NF-κB, thereby inhibiting the proinflammatory cytokine production by macrophages [18,19]. However, it is still unknown if inhibiting NOX2 activity in the context of dysfunctional AMPK in macrophages can inhibit inflammation by regulating proinflammatory cytokine production. In our recent study, we demonstrated the upregulation of DSS-induced intestinal inflammation in AMPKβ1 myeloid-deficient (AMPKβ1^LysM^) mice [4]. In this study, we investigated whether VAS2870 can inhibit DSS-induced intestinal inflammation in AMPKβ1^LysM^ mice. We found that VAS2870 ameliorated DSS-induced colitis in AMPKβ1^LysM^ and reversed the LPS-induced TLR4 and NOX2 expression, ROS production, p65 NF-κB nuclear translocation, and proinflammatory cytokine secretion in bone marrow-derived macrophages of AMPKβ1^fl/fl^ and AMPKβ1^LysM^ mice. Additionally, along with inhibiting LPS-induced proinflammatory cytokine secretion, VAS2870 reversed LPS-induced alterations in autophagy markers similar to sodium salicylate in RAW 264.7 cells. Overall, our study suggests that targeting NOX2 ameliorates colitis independent of AMPK activation which may render NOX2 a suitable target in the treatment of IBD and metabolic diseases. 

## 2. Materials and Methods

### 2.1. Mice

Age-matched (6–8 weeks old) AMPKβ1 myeloid-deficient mice were used. The generation of these mice was previously described in [4]. Briefly, gene-trap mice (C57BL/6N-Prkab1tm1a(KOMP)Wtsi) were initially crossed to FLPo recombinase-expressing mice (B6.Cg-Tg(Pgk1-flpo)10Sykr/J; JAX 011065). This removed the LacZ reporter and neomycin cassette, yielding mice with loxP sites flanking exon 2. To obtain AMPKβ1 myeloid-deficient mice, Prkab1 floxed mice were paired with mice expressing Cre recombinase under the control of the LysM promoter (B6.129P2-Lyz2tm1(cre)Ifo/J; JAX 004781). Mice were maintained by breeding floxed mice that were Cre-positive with floxed mice that were Cre-negative, such that comparisons were made between littermate animals. Hereafter, the floxed mice are expressed as AMPKβ1^fl/fl^, and AMPKβ1 myeloid-deficient mice are shown as AMPKβ1^LysM^. All experimental animal procedures followed the guidelines and principles of the Canadian Council of Animal Care and were approved by the Animal Care Committee at the University of Ottawa and McMaster University.

### 2.2. Reagents

DMEM high glucose was obtained from Hyclone (GE Healthcare Life Sciences, Logan, UT, USA). Fetal bovine serum (FBS) and penicillin/streptomycin were purchased from Invitrogen Life Technologies (Carlsbad, CA, USA), while Trypsin/EDTA was purchased from Clonetics, Inc. (Walkersville, MD, USA). Lipopolysaccharide (LPS), Sodium salicylate, and VAS2870 were purchased from Sigma-Aldrich (St. Louis, MO, USA). Phospho-AMPK-α (T172) (1:1000, Cat. #2535), AMPKα (1:1000, Cat. #5831), LC3 (1:1000, Cat. #12741), Beclin-1 (1:1000, Cat. #3495), Atg12-5 (1:1000, Cat. #4180), p62 (1:1000, Cat. #5114), and β-actin (1:1000, Cat. #4970) were obtained from Cell Signaling Technology, Inc. (Boston, MA, USA). NOX2 (1:1000, Cat. #ab129068), NF-κB p65 (1:1000, Cat. #ab16502), and Lamin B1 (1:1000, Cat. #ab65986) were purchased from Abcam (Cambridge, MA, USA).

### 2.3. Evaluation of DSS-Induced Colitis

Mice were divided into four groups, and the control group received only drinking water. DSS (mol wt. 36–54 kilodaltons: ICN Biomedicals Inc., Soho, OH, USA) was administered to the mice in the DSS, DSS + VAS2870, and DSS + Sodium salicylate groups via drinking water at 5% *w*/*v* for five days. Sodium salicylate (2 mg/kg) or VAS2870 (20 mg/kg) was administered intraperitoneally (i.p.). The records of average DSS consumption were recorded for each cage, daily throughout the experiment. All mice were sacrificed on the fifth day after DSS administration to evaluate the severity of colitis using previously published scoring systems [20]. Disease activity index (DAI) was quantified considering body weight loss, blood in feces, and stool consistency. Macroscopic scoring was performed immediately after the mice were sacrificed based on rectal bleeding, rectal prolapse, diarrhea, and colonic bleeding. Colonic histological damage was scored based on loss architecture, goblet cell depletion, crypt abscess, and inflammatory cell infiltration. Myeloperoxidase (MPO; an index of granulocyte infiltration and inflammation) activity was determined using a published protocol [20]. 

### 2.4. Cell Culture

The murine macrophage cell line RAW 246.7 (Passage 15–18), received from Dr. Dawn M. Bowdish’s lab, was cultured in Dulbecco’s Modified Eagles Medium supplemented with 10% FBS, 100 IU/mL of penicillin, and 100 μg/mL of streptomycin. Cells were maintained at 37 °C in 5% CO_2_. After the cells reached 70% confluency, they were split in a 1:3 ratio.

### 2.5. Isolation of Bone Marrow-Derived Macrophage

Bone marrow-derived macrophages were generated by slightly modifying the previously described method [21]. Briefly, AMPKβ1^fl/fl^ and AMPKβ1^LysM^ mice were euthanized to isolate the tibia and femur, and the end of the bones was cut off and placed into a sterile 0.5 mL microfuge tube with a hole in the end, punctured with an 18-gauge needle, which was then placed inside 1.5 mL microfuge tube. In total, 100 µL of DMEM was added to a 0.5 mL tube, and the bone marrow cells were collected by centrifuging the 1.5 mL tube at 2000 rpm for 4 min. The cells were resuspended in 100 mL DMEM supplemented with 10% FBS, 100 IU/mL of penicillin, and 100 μg/mL of streptomycin and plated in 6-well plates and placed in the incubator at 37 °C in 5% CO_2_. In total, 25 ng/mL of mouse macrophage colony-stimulating factor (M-CSF), purchased from R&D System Inc. (Minneapolis, MN, USA), was added to each well, and the cells were left to differentiate for 7 days. After the differentiation, the cells were treated as required, the supernatant was used for ELISA, and the cells were lysed for Western blotting.

### 2.6. Enzyme-Linked Immunosorbent Assay (ELISA)

Colon tissue samples were prepared as previously described [22] and supernatant from the cells was collected after the c treatment. Cytokine (IL-1β, IL-6, and TNF-α) levels in the supernatant of cells and tissue samples were determined using the ELISA kit provided by the R&D system (Minneapolis, MN, USA) and expressed in unit/mg of protein.

### 2.7. ROS Measurement

Intracellular ROS was measured using 2, ‘7’-dichlorofluorescein diacetate (DCF-DA), a cell-permeable fluorogenic probe, with slight modification, as described previously [23,24]. Briefly, RAW 246.7 (1 × 10^5^ cells/cm^2^) seeded in 6-well plates and 96-well black polystyrene flat-bottom plates were pretreated and treated with VAS2870 and SS for 1 h before treatment with LPSs for different periods. The cells were then washed with PBS and incubated with 10 μM DCF-DA for 30 min at 37 °C. After washing 3 times with PBS, the 96-well plates were placed in a SpectraMax M5 multi-detection reader (Molecular Devices, Sunnyvale, CA, USA) for fluorometric analysis using excitation and emission wavelengths of 488 and 520 nm, respectively, and images of the cells in 6-well plates were captured using a Nikon Eclipse 80i microscope and NIS-Element Basic Research imaging software. The fluorescence intensity was quantified using Image J.

### 2.8. Western Blotting

An NE-PER nuclear and cytoplasmic extraction reagent kit (#78833, Thermo Scientific, Rockford, IL, USA) was used to extract cytoplasmic and nuclear protein extracts, as described previously [25]. Whole-cell lysates were extracted using radio immunoprecipitation assay (RIPA) buffer containing 1× protease and phosphatase inhibitor cocktail and centrifuged at 12,000 rpm for 10 min. The supernatants were collected, and protein concentration was determined using the BCA protein assay kit (Pierce, Rockford, IL, USA) and DC protein assay kit (Bio-Rad, Mississauga, ON, Canada). The protein samples were separated using sodium dodecyl sulfate–polyacrylamide gel electrophoresis (SDS-PAGE) and then electrophoretically transferred onto nitrocellulose or polyvinylidene difluoride (PVDF) membranes. The membranes were incubated with 5% bovine serum albumin (BSA) in 1× Tris-buffered saline (TBS) and Tween 20 (TBS-T) at room temperature for 1 h and then probed with primary antibodies overnight at 4 °C. The membranes were then washed three times with 1× TBST followed by incubation with the corresponding secondary antibodies for 1 h at room temperature. Immunodetection was performed by visualization of the membrane using a chemiluminescent reagent (Thermo Scientific, Rockford, IL, USA) and by exposure to a luminescent image analyzer, LAS-4000 mini (Fujifilm, Tokyo, Japan).

### 2.9. Immunohistochemistry

Mice colon tissues fixed in 10% buffered formalin and embedded in paraffin were stained for NOX2. The tissue sections were deparaffinized with Xylene (catalog no. 9800-1, Caledon) and rehydrated sequentially in graded concentrations of ethanol. After heat-induced epitope retrieval, tissues were blocked with 3% bovine serum albumin and incubated with a rabbit monoclonal NOX2/gp91phox antibody (1:200; catalog no. ab129068, Abcam) for 1 h at room temperature. The sections were washed with phosphate-buffered saline/0.5% Tween 20 and then incubated with EnVision (horseradish peroxidase-coupled anti-rabbit secondary reagent; DakoCytomation, catalog no. K4003, Dako) for 30 min. The development of the sections was performed using a 3,3′-diaminobenzidine solution (SIGMAFAST, catalog no. 079K8208, Sigma-Aldrich) and counterstained with Mayer’s hematoxylin solution (catalog no. MHS1, Sigma-Aldrich). The sections were finally visualized using a Nikon Eclipse 80i microscope (Nikon Instruments Inc. Melville, NY, USA). 

### 2.10. Statistical Analysis

Data are expressed as the mean ± standard error of the mean (S.E.M). Where appropriate, a comparison with two groups was made using Student’s *t*-test for unpaired data and Student’s one-way ANOVA in Graph Pad Prism ver. 5.0 (San Diego, CA, USA) to determine the significance of intergroup differences. A *p*-value < 0.05 was considered statistically significant.

## 3. Results

### 3.1. VAS2870 Inhibits DSS-Induced Colitis in AMPKβ1^LysM^ Mice

**Figure 1:**
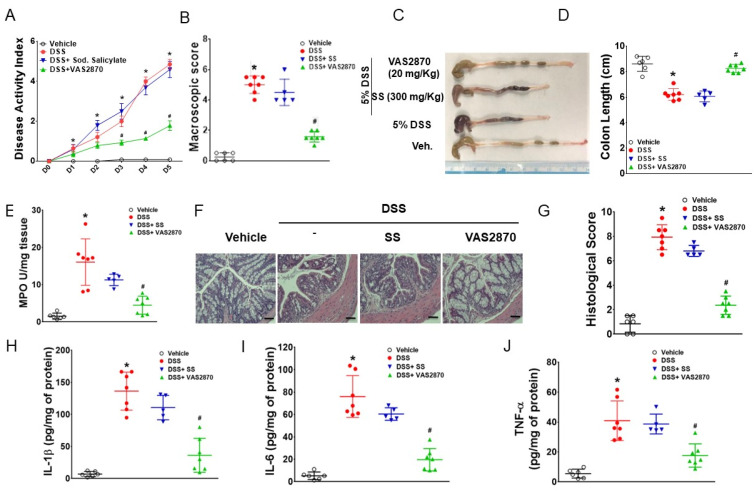
VAS2870 inhibited DSS-induced colitis in AMPKβ1^LysM^ mice but sodium salicylate did not. Sodium salicylate and VAS2870 were injected intraperitoneally from the same day of administration of 5% DSS in drinking water ad libitum in AMPKβ1^LysM^ mice for 5 days, and mice were killed on day 5 post-DSS to assess inflammation in mice by analyzing (**A**) DAI, (**B**) macroscopic score, (**C**) representative colons, (**D**) colon length, (**E**) MPO activity, (**F**) representative micrographs of hematoxylin and eosin-stained colon cross-sections on day 5 post-DSS; bar = 100 μm, and (**G**) histological scores. (**H**) IL-1β, (**I**) IL-6, and (**J**) TNF-α levels were measured using ELISA. Data represent ± SEM (*n* = 5). * *p* < 0.05, compared with water-receiving mice; # *p* < 0.05, compared with DSS-receiving AMPKβ1^LysM^ mice.

The colon tissues of DSS-treated AMPKβ1^LysM^ mice showed a significant increase in NOX2 expression compared to DSS-treated AMPKβ1^fl/fl^ mice (Figure S1). Therefore, to investigate whether the inhibition of NOX2 ameliorates DSS-induced intestinal inflammation, mice were treated with VAS2870 (20 mg/kg) or sodium salicylate; SS (300 mg/kg) intraperitoneally (i.p.) from day 0 of 5% DSS administration. Treatment with VAS2870 ameliorated DSS-induced colitis with a reduction in DAI, MPO levels, macroscopic and histological scores, secretion of proinflammatory cytokines (TNF-α, IL-6, and IL-1β), and an increase in colon length, which were not affected by SS treatment (Figure 1A–I). Additionally, the DSS-induced expression of NOX2 was inhibited by VAS2870 but not by SS in mice colon tissue as determined using immunohistochemistry (Figure S2). There was no effect of SS on DSS-induced inflammation in AMPKβ1^LysM^ mice, which aligns with our previous study [4]. These data indicate that the inhibition of NOX2 ameliorates colitis in mice even in the absence of macrophage AMPK activity.

### 3.2. NOX2-Derived ROS Regulates the Expression of TLR4, NOX2, IL-1β, IL-6, and TNF-α in LPS-Induced Bone Marrow-Derived Macrophages with Impaired AMPK

**Figure 2:**
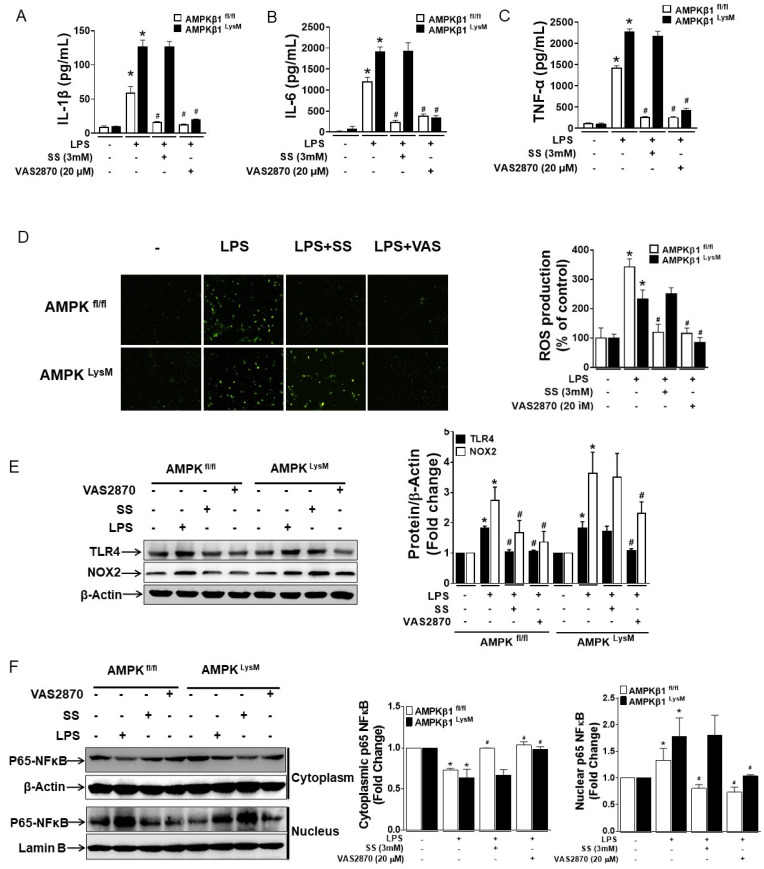
NOX2 regulates LPS-induced inflammatory response in both AMPKβ1^fl/fl^ and AMPKβ1^LysM^ mice. BMDMs from AMPKβ1^fl/fl^ and AMPKβ1^LysM^ mice were pre-treated with either 3 mM SS or 20 µM VAS2870 for 1 h before the treatment with LPSs for 24 h, and the supernatant was taken to measure the levels of (**A**) IL-1β, (**B**) IL-6, and (**C**) TNF-α. (**D**) The cells were then incubated with 10 μM DCF-DA for 30 min at 37 °C. After washing 3 times with PBS, the fluorescence image was captured using a Nikon Eclipse 80i microscope and were analyzed using ImageJ. (**E**) The cells were similarly treated and harvested for the extraction of protein, and the proteins were analyzed for the expression of NOX2 and TLR4 using Western blot. (**F**) BMDM cells from AMPKβ1^fl/fl^ and AMPKβ1^LysM^ mice were pre-treated with either SS or VAS2870 for 1 h before the treatment with LPSs for 30 min, and the cells were harvested, the cytoplasmic and nuclear protein were isolated, and the expression of p65 NFκB was analyzed in the cytoplasm and nuclear fragments. The bar graph represents the quantitative data. * *p* < 0.05, compared with untreated cells; # *p* < 0.05, compared with LPS-treated cells.

As the inhibition of NOX2 in DSS-treated macrophage AMPK-deficient mice prompted reduced inflammation, it was necessary to investigate whether NOX2 regulates LPS-induced pro-inflammatory cytokine secretion in macrophages. Therefore, BMDMs, which showed a similar response to the LPSs as residential colonic macrophages, were isolated from AMPKβ1^fl/fl^ and AMPKβ1^LysM^ mice [4]. BMDMs were pretreated with either VAS2870 or SS for 1 h before treatment with LPSs for the indicated time. Treatment with both VAS2870 and SS inhibited LPS-induced IL-1β, IL-6, and TNF-α secretion in AMPKβ1^fl/fl^ BMDMs, while only VAS2870 inhibited the secretion in both AMPKβ1^fl/fl^ and AMPKβ1^LysM^ BMDMs (Figure 2A–C). Likewise, LPS-induced ROS production, TLR4, and NOX2 expressions were inhibited in both AMPKβ1^fl/fl^ and AMPKβ1^LysM^ BMDMs by VAS2870, whereas SS was effective in AMPKβ1^fl/fl^ BMDMs.

NF-κB, a redox-sensitive transcriptional factor that is reported to regulate pro-inflammatory cytokines such as IL-1β, IL-6, and TNF-α, has also been confirmed to regulate the expression of TLR-4 and NOX2 [26,27]. Furthermore, the BMDM cells were harvested to separate the cytosolic and the nuclear proteins to investigate whether VAS2870 exerts its effect in LPS-treated BMDMs by inhibiting the nuclear translocation of p65 NFκB. We observed that LPS-induced nuclear translocation of p65 NFκB in both AMPKβ1^fl/fl^ and AMPKβ1^LysM^ BMDMs was reversed by VAS2870, but SS reversed the translocation only in AMPKβ1^fl/fl^ BMDMs (Figure 2D–F). These findings suggest that during the deregulation of AMPK, the inhibition of NOX2 activity by VAS2870 in macrophages inhibits the production of ROS, thereby blocking the nuclear translocation of p65 NFκB, suppressing the secretion of proinflammatory cytokines as well as the expression of TLR4 and NOX2. 

### 3.3. Both SS and VAS2870 Reverse LPS-Induced Effects in RAW 246.7 Targeting ROS/AMPK Axis 

**Figure 3:**
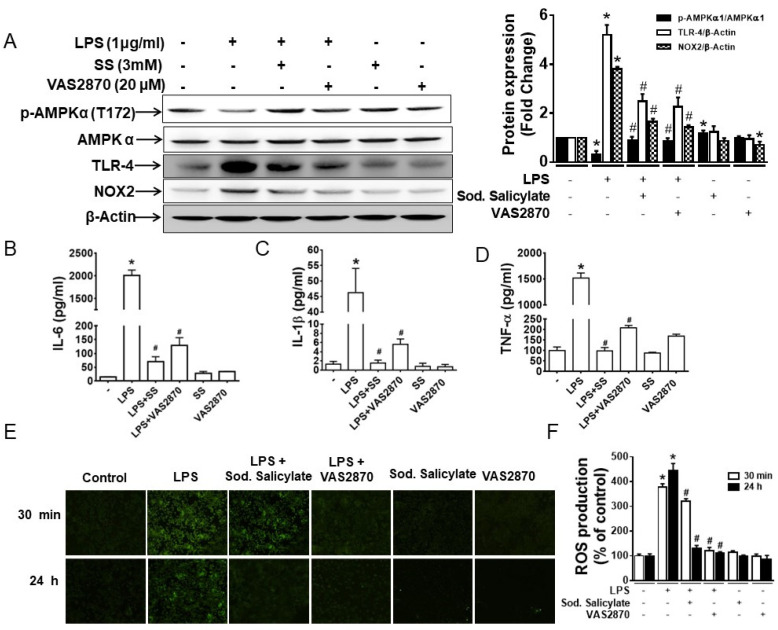
Inhibition of AMPK activation and NOX2 expression suppresses LPS-induced response in macrophages. RAW 264.7 macrophage cells were pretreated with SS and VAS2870 for 1 h before treatment with LPSs for 24 h. Cells were harvested, and the supernatant was collected. (**A**) The extracted total protein from the cells was analyzed for expression of phospho-AMPKα1, AMPKα1, TLR4, and NOX2. The bar graph represents the quantitative data. * *p* < 0.05, compared with untreated cells; # *p* < 0.05, compared with LPS-treated cells. The supernatants were analyzed for (**B**) IL-6, (**C**) IL-1β, and (**D**) TNF-α. * *p* < 0.05, compared with untreated cells; # *p* < 0.05, compared with LPS-treated cells. RAW 264.7 macrophage cells were pretreated with SS and VAS2870 for 1 h before treatment with LPSs for 30 min and 24 h in 6-well plates and 96-well black polystyrene flat-bottom plates. The cells were then washed with PBS and incubated with 10 μM DCF-DA for 30 min at 37 °C. After washing three times with PBS, fluorescent images in (**E**) 6-well plates were captured using a Nikon Eclipse 80i microscope and (**F**) the 96-well plate was placed in a SpectraMax M5 multi-detection reader for fluorometric analysis using excitation and emission wavelengths of 488 and 520 nm. * *p* < 0.05, compared with untreated cells; # *p* < 0.05, compared with LPS-treated cells.

To further elucidate the mechanism by which VAS2870 inhibits the effect of LPSs, RAW 246.7 cells were pre-treated with SS and VAS2870 for 1 h before the treatment with LPSs for 24 h. The total protein extracts were subjected to Western blotting to analyze the expressions of p-AMPK, AMPK, NOX2, and TLR4. LPS-induced dephosphorylation of AMPK and increased expression of TLR4 and NOX2 were reversed by both SS and VAS2870 (Figure 3A). Similarly, LPS-induced secretion of IL-1β, IL-6, and TNF-α was also suppressed by VAS2870 and SS (Figure 3B–D). The finding suggests that both VAS2870 and SS eventually inhibit NOX2 activity to suppress the secretion of IL-1β, IL-6, and TNF-α.

The anti-inflammatory activity of salicylates has been partly associated with their antioxidant effect. To comprehend the differential mechanism by which VAS2870 and SS inhibit the LPS-induced ROS production in macrophages, RAW 246.7 cells were pre-treated with SS and VAS2870 for 1 h and treated with LPSs for 30 min and 24 h, and ROS production was analyzed at different time points. At 30 min, SS slightly inhibited LPS-induced ROS production, while VAS2870 completely blocked it. At 24 h, both SS and VAS2870 were able to block the LPS-induced ROS production (Figure 3E,F). The data indicate that SS weakly inhibits ROS production at 30 min by the virtue of its antioxidant properties, whereas at 24 h, SS inhibits ROS production by activating AMPK and subsequently inhibiting NOX2 expression. Overall, the findings indicate that VAS2870 directly inhibits NOX2 expression and suppresses LPS-induced ROS production, thereby inhibiting the inflammation, whereas SS indirectly suppresses NOX2 expression by phosphorylation and activation of AMPK, resulting in the inhibition of LPS-induced ROS production.

### 3.4. Inhibition of NOX2 Activity Rescues LPS-Induced Impaired Autophagy

**Figure 4:**
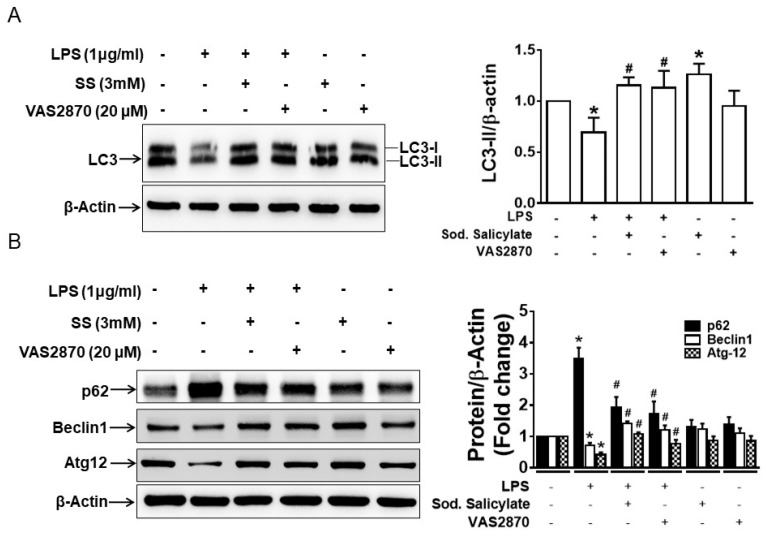
Inhibition of NOX2 expression and activation of AMPK reversed LPS-induced impaired autophagy in macrophages during inflammation. RAW 264.7 macrophage cells were pretreated with SS and VAS2870 before treatment with LPS for 24 h. (**A**) Proteins extracted from the cells were analyzed for the expression of (**A**) LC3 and (**B**) p62, Beclin-1, and Atg-12. The bar graphs represent the quantitated data. * *p* < 0.05, compared with untreated cells and # *p* < 0.05, compared with LPS-treated cells.

Impaired autophagy has been highlighted as an important contributing factor to increasing inflammation during colitis [28,29]. In our previous study, we demonstrated that SS reversed the LPS-induced downregulated expression of autophagy markers (LC-3, Beclin-1, and Atg-12) and upregulated the expression of p62 in macrophages [4]. Therefore, to examine whether VAS2870 has any effect on LPS-induced impaired autophagy, RAW 246.7 cells were pre-treated with SS and VAS2870 for 1 h and treated with LPSs for 24 h. Cells were harvested, and the total protein extracts were analyzed for the expression of LC-3, p62, Beclin-1, and Atg-12. Here, treatment with VAS2870 reversed the LPS-induced impaired autophagy similarly to SS (Figure 4A,B).

These data suggest an important role of both NOX2 and AMPK in regulating autophagy to limit inflammation.

## 4. Discussion

To our knowledge, this study demonstrates for the first time that the inhibition of NOX2 by VAS2870 ameliorates macroscopic and histological scores, MPO levels, reduces colon length, and suppresses the secretion IL-1β, IL-6, and TNF-α independently of AMPK activation in DSS-induced colitis. These effects were largely dependent on the inhibitory action of VAS2870 on macrophage NOX2. The effects of activation of AMPK by SS and the inhibition of NOX2 using VAS2870 were similar in LPS-treated AMPKβ1^fl/fl^ BMDMs, whereas LPS-induced effects in AMPKβ1^LysM^ BMDMs were inhibited by VAS2870, indicating that functional AMPK in macrophages regulates the expression of NOX2 and ROS production upon inflammatory insult, as observed when stimulated with LPSs. In our recent study, activation of macrophage AMPK using SS ameliorated DSS-induced intestinal inflammation in mice [4], whereas, in the case of dysfunctional AMPK, our present study suggests the inhibition of NOX2 as a strong alternative. The significant increase in NOX2 expression in the colon tissue of AMPKβ1^LysM^ compared to AMPKβ1^fl/fl^, as observed in Figure S1, suggests a regulatory role of AMPK in the expression of NOX2.

The regulation of NOX2 by AMPK in the cardiovascular system has previously been reported. Impaired AMPK activity due to the deletion of AMPKα2 resulted in the aberrant expression and activation of NOX2, leading to endothelial dysfunction, which was abolished with the inhibition of NOX2 activity [30]. Furthermore, phorbol ester-induced reduction of AMPK phosphorylation was observed to be dependent on NOX2-derived ROS production in HT29 colonic epithelial cells [24]. Various studies suggest a key role of serotonin (5-HT) in the pathogenesis of colitis and NOX2-derived ROS as one of the mediators in this process [23,31]. NOX2-derived ROS mediated commensal E. coli-induced upregulation of TLR2/TLR4, IL-8, and ICAM-1 in HT29 and CCD841 colon epithelial cells. Furthermore, mice colon inoculated with commensal E. coli and high 5-HT that induced fatal inflammation in mice showed an increased expression of NOX2 and TLR2/TLR4 [25]. In our present study, LPS-induced ROS in AMPKβ1^fl/fl^ BMDMs is inhibited by both SS and VS2870, whereas in AMPKβ1^LysM^ BMDMs, only VAS2870 inhibited ROS production. This finding along with the data showing differential inhibitory activity of SS on LPS-induced ROS production in macrophages at a different time points suggests that SS requires functional AMPK to inhibit the LPS-induced NOX2-derived ROS. Here, NOX2-derived ROS mediates the dephosphorylation of AMPK, nuclear translocation of p65 NFκB, and secretion of proinflammatory cytokines, along with the increase in the expression of TLR-4 and NOX2 in LPS-treated macrophages. Overall, the data indicate an inverse correlation of NOX2 expression and AMPK phosphorylation, suggesting a feedback loop between AMPK and NOX in macrophages. However, targeting NOX2 can also regulate inflammation independent of AMPK activation. We previously illustrated that DSS-induced inflammation and colitis in AMPKβ1-deficient mice may result from impaired autophagy in macrophages [4]. Additionally, a dysregulated autophagic process and polymorphism in autophagy genes contribute to the development of inflammatory bowel disease [28,32]. Here, we found that LPS-induced impaired autophagy in macrophages was prevented by VAS2870 and SS. Both SS and VAS2870 reversed the LPS-induced suppressed expression of autophagy markers (LC-3, Beclin-1, and Atg-12) and increased the expression of p62 in macrophages.

**Figure 5:**
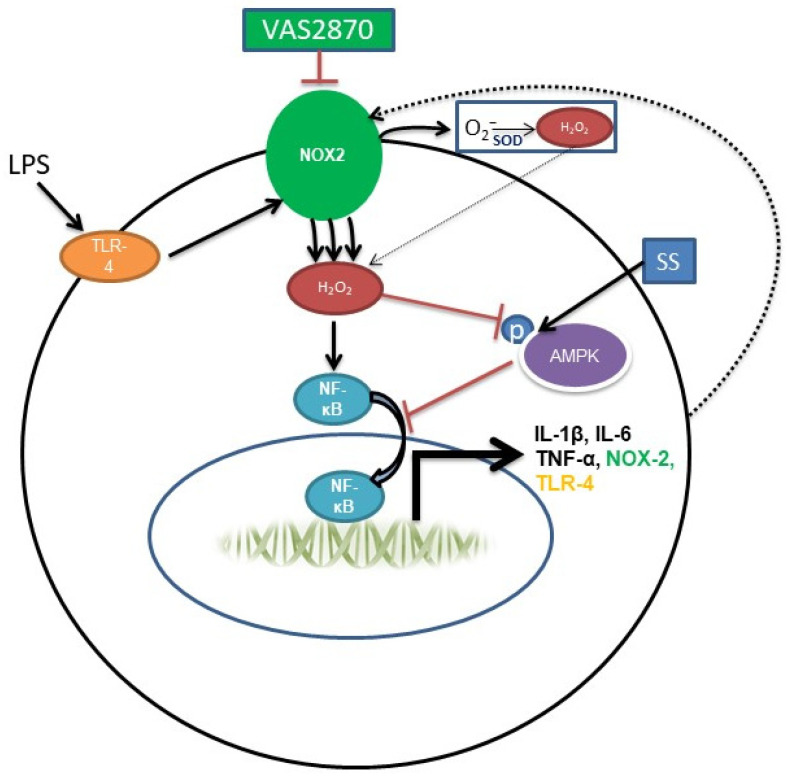
LPS stimulate NOX2 in macrophages by activating TLR4, which enhances ROS production that induces various proinflammatory cytokines, such as IL-1β, IL-6, and TNF-α, along with NOX2 itself (via transcription factors such as NF-κB). AMPK in its active form inhibits the induction of cytokines and NOX-2 to counteract the insults. In a macrophage with dysfunctional AMPK, this balance is disturbed, leading to increased cytokine secretion and prolonged NOX-2 activation as well as the aggravation of inflammatory reaction.

Previously published studies have indicated that inhibition of NOX activity and ROS signaling in macrophages to control their response has beneficial effects in the treatment of different immune–inflammatory diseases [33,34,35,36]. NOX1 is reported to be highly expressed in colon tissue, but its role in colitis is still unclear. Previous studies have illustrated alterations in NOX1 expression [18,19]; however, the vast majority suggest that NOX2-derived ROS regulates inflammation during colitis [5,19,23,25]. Furthermore, a significant increase in the expression of NOX2 in lamina propria mononuclear cells (LPMCs) of patients with Crohn’s disease and ulcerative colitis compared to a healthy control has been reported [5]. Likewise, the use of metformin, which is known to activate AMPK, is associated with a lower risk of IBD in patients with Type 2 diabetes mellitus [37]. Inhibiting NOX2-derived ROS production using pan-NOX inhibitors or antioxidants has also been shown to alleviate the inflammatory response in both TNBS and DSS models that display characteristics of CD and UC, respectively [5,23,38]. These findings, along with our own animal work, provide an important milestone in the development of therapies to minimize oxidative stress due to persistent ROS production by predominant contributors, such as NOX, that may deactivate AMPK and alters its control over the inflammatory signals (Figure 5). As noted previously, the limited efficacy of NOX inhibitors, including VAS2870, in thoroughly inhibiting NOX bioactivities and signals to normal levels warrants the development of more potent inhibitors [5]. Irreversible inhibition or defects in the regulation of NOX2 is associated with chronic granulomatous disease that urges the development of potent and reversible NOX2 inhibitors and examining their long-term effects in IBD patients [39].

Overall, our study suggests that inhibition of NOX2 may be an important alternative strategy, independent of AMPK, to manage intestinal inflammation and conditions such as inflammatory bowel disease. 

## Data Availability

All data supporting the results in this study is available in this article.

## Supplementary Materials

The following supporting information can be downloaded at https://www.mdpi.com/article/10.3390/biomedicines11051443/s1, Figure S1: NOX2 expression increased significantly in DSS-treated AMPKβ1^LysM^ mice. The protein extracted from the colonic tissue of control and DSS-treated AMPK β1fl/fl and AMPK β1^LysM^ mice were analyzed for NOX2 by Western blot. The bar graph represents the quantitative data. * *p* < 0.05, compared with untreated mice; # *p* < 0.05, compared with DSS-treated AMPK β1^fl/fl^ mice; Figure S2: DSS-induced NOX2 expression is inhibited by VAS2870 but not by SS in colon tissue of AMPKβ1^LysM^ mice, bar = 100 μm.
