# Supplementary material for: Inhibition of NADPH Oxidase (NOX) 2 Mitigates Colitis in Mice with Impaired Macrophage AMPK Function
Source: Biomedicines. 2023 May 14;11(5):1443. doi: 10.3390/biomedicines11051443 (PMC10216132; doi:10.3390/biomedicines11051443)
Supplement: Supplementary file 1 [file biomedicines-11-01443-s001.zip › biomedicines-2361050-supplementary.pdf]

Supplementary Materials

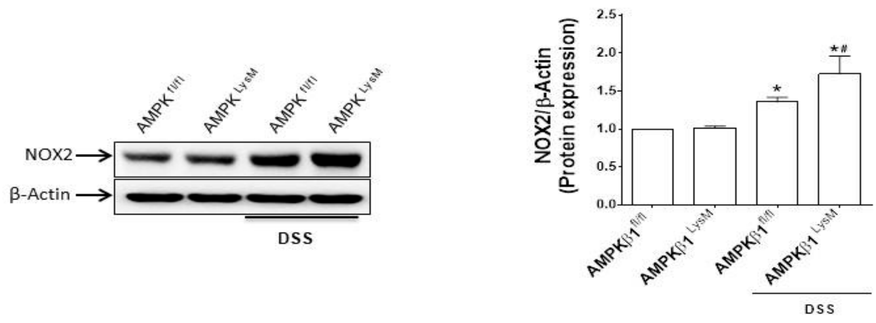

Figure S1. NOX2 expression increased significantly in DSS-treated AMPKβ1LysM mice. The protein extracted from the colonic tissue of control and DSS-treated AMPK β1fl/fl and AMPK β1LysM mice were analyzed for NOX2 by western blot. The bar graph represents the quantitative data. * $p < 0.05$, compared with untreated mice; # $p < 0.05$, compared with DSS-treated AMPK β1fl/fl mice.

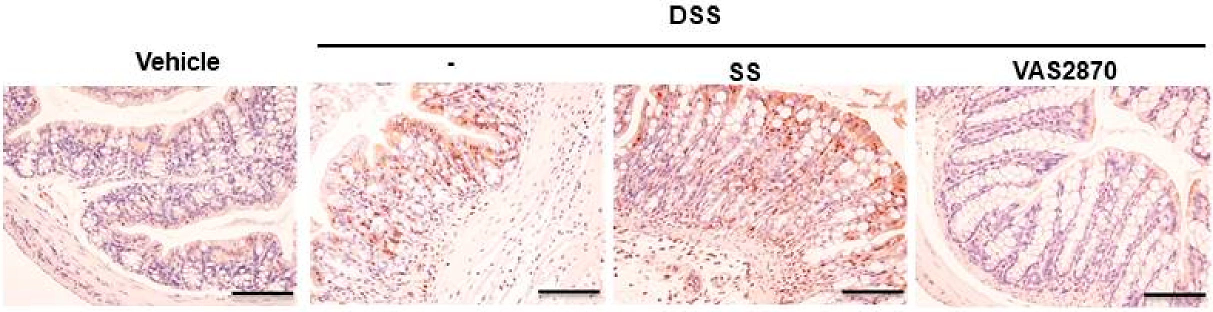

Figure S2. DSS-induced NOX2 expression is inhibited by VAS2870 but not by SS in colon tissue of AMPKβ1LysM mice, bar = 100 μm.
